# Supplementary material for: Low capacity for molecular detection of Alphaviruses other than Chikungunya virus in 23 European laboratories, March 2022
Source: PLoS One. 2025 Feb 27;20(2):e0318602. doi: 10.1371/journal.pone.0318602 (PMC11867335; doi:10.1371/journal.pone.0318602)
Supplement: S2 Table — (DOCX) [file pone.0318602.s002.docx]

**Supplementary Table 1. Performance of all assays used in the 2022 EVD-LabNet EQA on molecular detection of alphaviruses.**

| **Assay design** | **Method** | **Result level** | **Reference  or manufac-turer** | **Target** | **RRV** | **SINV** | **WEEV** | **VEEV** | **EEEV** | **BFV** | **MAYV** | **ONNV** | **CHIKV-Asian** | **CHIKV- West African** | **CHIKV-ECSA** | **Negative samples** | **False positive (FP)** | **False negative (FN)** | **Incon-clusive** | **Correct entries, n (%)** |
| --- | --- | --- | --- | --- | --- | --- | --- | --- | --- | --- | --- | --- | --- | --- | --- | --- | --- | --- | --- | --- |
| Pan-alphavirus RT-PCR | In-house RT-qPCR | Genus | Giry et al.(61) ^a^ | NSP4 | 2/2 | 2/2 | 2/2 | 1/2 | 2/2 | 2/2 | 2/2 | 2/2 | 2/2 | 2/2 | 2/2 | 5/8 | 3 | 1 | - | 26/30 (87%) |
|  | In-house conventional RT-PCR followed by sequencing | Species | Sánchez-Seco et al.(62) | NSP4 | 4/4 | 4/4 | 3/4 | 2/4 | 2/4 | 2/4 | 3/4 | 4/4 | 4/4 | 4/4 | 4/4 | 14/16 | 1 | 7 | 2 | 50/60 (83%) |
|  |  |  | Grywna et al.(63) | NSP4 | 3/3 | 2/3 | 2/3 | 0/3 | 1/3 | 3/3 | 3/3 | 3/3 | 3/3 | 3/3 | 3/3 | 12/12 | - | 7 | - | 38/45 (84%) |
|  |  |  | Eshoo et al.(64) ^b^ | NSP4 | 1/1 | 2/2 | 1/2 | 2/2 | 1/2 | 1/1 | 1/1 | 1/1 | 1/1 | 1/1 | 1/1 | 8/8 | - | 2 | - | 21/23 (91%) |
|  |  |  | Eshoo et al.(64) ^b^ | NSP1 | - | 0/1 | 1/1 | 1/1 | 1/1 | - | - | - | - | - | - | 4/4 | - | 1 | - | 7/8 (88%) |
|  |  |  | de Morais Bronzoni et al.(65) | NSP1 | 1/1 | 1/1 | 1/1 | 0/1 | 0/1 | 1/1 | 0/1 | 0/1 | 1/1 | 1/1 | 0/1 | 1/4 | 7 | 1 | - | 7/15 (47%) |
|  |  |  | Hermanns et al.(66) | NSP4 | 1/1 | 1/1 | 1/1 | 1/1 | 1/1 | 1/1 | 1/1 | 1/1 | 1/1 | 1/1 | 1/1 | 4/4 | - | - | - | 15/15 (100%) |
|  |  |  | Not published (a) | Unknown | 1/1 | 0/1 | 0/1 | 0/1 | 0/1 | 0/1 | 0/1 | 1/1 | 1/1 | 1/1 | 1/1 | 4/4 | 1 | 5 | - | 9/15 (60%) |
| WTA | In-house | Species | Rosenstierne et al.(67) | Unknown | 1/1 | 1/1 | 1/1 | 1/1 | 1/1 | 1/1 | 1/1 | 1/1 | 1/1 | 1/1 | 1/1 | 3/4 | 1 | - | - | 14/15 (93%) |
| HTS | N/A | Species | N/A | N/A | 1/1 | 1/1 | 1/1 | 1/1 | 1/1 | 1/1 | 1/1 | 1/1 | 1/1 | 1/1 | 1/1 | 4/4 | - | - | - | 15/15 (100%) |

| **Assay design** | **Method** | **Result level** | **Reference  or manufac-turer** | **Target** | **RRV** | **SINV** | **WEEV** | **VEEV** | **EEEV** | **BFV** | **MAYV** | **ONNV** | **CHIKV-Asian** | **CHIKV- West African** | **CHIKV-ECSA** | **Negative samples** | **False positive (FP)** | **False negative (FN)** | **Incon-clusive** | **Correct entries, n (%)** |
| --- | --- | --- | --- | --- | --- | --- | --- | --- | --- | --- | --- | --- | --- | --- | --- | --- | --- | --- | --- | --- |
| RRV-specific  RT-PCR | In-house  RT-qPCR | Species | Inglis et al.(68) | E1 | 1/1 | 1/1 | 1/1 | 1/1 | 1/1 | 1/1 | 1/1 | 1/1 | 1/1 | 1/1 | 1/1 | 4/4 | - | - | - | 15/15 (100%) |
|  |  |  | Kizu et al.(69) | E2 | 1/1 | 1/1 | 1/1 | 1/1 | 1/1 | 1/1 | 0/1 | 1/1 | 1/1 | 1/1 | 1/1 | 4/4 | - | - | 1 | 14/15 (93%) |
|  |  |  | Not published (b) | E2 | 1/1 | 1/1 | 1/1 | 1/1 | 1/1 | 1/1 | 1/1 | 1/1 | 1/1 | 1/1 | 1/1 | 4/4 | - | - | - | 15/15 (100%) |
|  |  |  | Not published (c) | NSP1 | 1/1 | 1/1 | 1/1 | 1/1 | 1/1 | 1/1 | 1/1 | 1/1 | 1/1 | 1/1 | 1/1 | 4/4 | - | - | - | 15/15 (100%) |
|  |  |  | Not published (d) | NSP2/E2 | 1/1 | 1/1 | 1/1 | 1/1 | 1/1 | 1/1 | 1/1 | 1/1 | 1/1 | 1/1 | 1/1 | 4/4 | - | - | - | 15/15 (100%) |
|  |  |  | Not published (e) | NSP3 | 1/1 | 1/1 | 1/1 | 1/1 | 1/1 | 1/1 | 1/1 | 1/1 | 1/1 | 1/1 | 1/1 | 4/4 | - | - | - | 15/15 (100%) |
| SINV-specific  RT-PCR | In-house  RT-qPCR | Species | Jöst et al.(70) | NSP1 | 2/2 | 2/2 | 2/2 | 2/2 | 2/2 | 2/2 | 2/2 | 2/2 | 2/2 | 2/2 | 2/2 | 8/8 | - | - | - | 30/30 (100%) |
|  |  |  | Sane et al.(71) | NSP1 | 1/1 | 1/1 | 1/1 | 1/1 | 1/1 | 1/1 | 1/1 | 1/1 | 1/1 | 1/1 | 1/1 | 4/4 | - | - | - | 15/15 (100%) |
|  |  |  | Not published (f) | NSP1/E1 | 1/1 | 1/1 | 1/1 | 1/1 | 1/1 | 1/1 | 1/1 | 1/1 | 1/1 | 1/1 | 1/1 | 4/4 | - | - | - | 15/15 (100%) |
|  |  |  | Not published (g) | NSP3 | 1/1 | 1/1 | 1/1 | 1/1 | 1/1 | 1/1 | 1/1 | 1/1 | 1/1 | 1/1 | 1/1 | 4/4 | - | - | - | 15/15 (100%)5/5 (100%) |
|  |  |  | Not published (h) | Unknown | 1/1 | 0/1 | 1/1 | 0/1 | 1/1 | 1/1 | 1/1 | 1/1 | 1/1 | 1/1 | 1/1 | 4/4 | 1 | 1 | - | 13/15 (87%) |

| **Assay design** | **Method** | **Result level** | **Reference  or manufac-turer** | **Target** | **RRV** | **SINV** | **WEEV** | **VEEV** | **EEEV** | **BFV** | **MAYV** | **ONNV** | **CHIKV-Asian** | **CHIKV- West African** | **CHIKV-ECSA** | **Negative samples** | **False positive (FP)** | **False negative (FN)** | **Incon-clusive** | **Correct entries, n (%)** |
| --- | --- | --- | --- | --- | --- | --- | --- | --- | --- | --- | --- | --- | --- | --- | --- | --- | --- | --- | --- | --- |
| WEEV-specific  RT-PCR | In-house  RT-qPCR | Species | Kang et al.(59) | E3 | 2/2 | 2/2 | 2/2 | 2/2 | 2/2 | 2/2 | 2/2 | 2/2 | 2/2 | 2/2 | 2/2 | 8/8 | - | - | - | 30/30  (100%) |
|  |  |  | Lambert et al.(72) | E1 | 1/1 | 1/1 | 1/1 | 1/1 | 1/1 | 1/1 | 1/1 | 1/1 | 1/1 | 1/1 | 1/1 | 4/4 | - | - | - | 15/15 (100%) |
|  |  |  | Not published (i) | C/NSP1 | 1/1 | 1/1 | 1/1 | 1/1 | 1/1 | 1/1 | 1/1 | 1/1 | 1/1 | 1/1 | 1/1 | 4/4 | - | - | - | 15/15 (100%) |
|  | Commercial  RT-qPCR |  | Filmarray BioThreat Panel, BioFire Defense | Unknown | 1/1 | 1/1 | 1/1 | 1/1 | 1/1 | 1/1 | 1/1 | 1/1 | 1/1 | 1/1 | 1/1 | 4/4 | - | - | - | 15/15 (100%) |
| VEEV-specific PCR | In-house RT-qPCR | Species | Vina-Rodriguez(60) | NSP1 | 2/2 | 2/2 | 2/2 | 2/2 | 2/2 | 2/2 | 2/2 | 2/2 | 2/2 | 2/2 | 2/2 | 8/8 | - | - | - | 30/30  (100%) |
|  |  |  | Not published (j) | NSP1 | 1/1 | 1/1 | 1/1 | 1/1 | 1/1 | 1/1 | 1/1 | 1/1 | 1/1 | 1/1 | 1/1 | 4/4 | - | - | - | 15/15 (100%) |
|  | Commercial RT-qPCR |  | Filmarray BioThreat Panel, BioFire Defense | Unknown | 1/1 | 1/1 | 1/1 | 1/1 | 1/1 | 1/1 | 1/1 | 1/1 | 1/1 | 1/1 | 1/1 | 4/4 | - | - | - | 15/15 (100%) |
| EEEV-specific PCR | In-house RT-qPCR | Species | Kang et al.(59) | NSP3 | 2/2 | 2/2 | 2/2 | 2/2 | 2/2 | 2/2 | 2/2 | 2/2 | 2/2 | 2/2 | 2/2 | 8/8 | - | - | - | 30/30  (100%) |
|  |  |  | Lambert et al.(72) | E2 | 1/1 | 1/1 | 1/1 | 1/1 | 1/1 | 1/1 | 1/1 | 1/1 | 1/1 | 1/1 | 1/1 | 4/4 | - | - | - | 15/15 (100%) |
|  |  |  | Armstrong et al.(73) | C/E2 | 1/1 | 1/1 | 1/1 | 1/1 | 1/1 | 1/1 | 1/1 | 1/1 | 1/1 | 1/1 | 1/1 | 4/4 | - | - | - | 15/15 (100%) |
|  |  |  | Not published (k) | E1 | 1/1 | 1/1 | 1/1 | 1/1 | 1/1 | 1/1 | 1/1 | 1/1 | 1/1 | 1/1 | 1/1 | 4/4 | - | - | - | 15/15 (100%) |
|  | Commercial RT-qPCR |  | Filmarray BioThreat Panel, BioFire Defense | Unknown | 1/1 | 1/1 | 1/1 | 1/1 | 1/1 | 1/1 | 1/1 | 1/1 | 1/1 | 1/1 | 1/1 | 4/4 | - | - | - | 15/15 (100%) |

| **Assay design** | **Method** | **Result level** | **Reference  or manufac-turer** | **Target** | **RRV** | **SINV** | **WEEV** | **VEEV** | **EEEV** | **BFV** | **MAYV** | **ONNV** | **CHIKV-Asian** | **CHIKV- West African** | **CHIKV-ECSA** | **Negative samples** | **False positive (FP)** | **False negative (FN)** | **Incon-clusive** | **Correct entries, n (%)** |
| --- | --- | --- | --- | --- | --- | --- | --- | --- | --- | --- | --- | --- | --- | --- | --- | --- | --- | --- | --- | --- |
| BFV-specific PCR | In-house RT-qPCR | Species | Inglis et al.(68) | NSP4 | 1/1 | 1/1 | 1/1 | 1/1 | 1/1 | 1/1 | 1/1 | 1/1 | 1/1 | 1/1 | 1/1 | 4/4 | - | - | - | 15/15 (100%) |
|  |  |  | Not published (l) | NSP1/E2 | 1/1 | 1/1 | 1/1 | 1/1 | 1/1 | 1/1 | 1/1 | 1/1 | 1/1 | 1/1 | 1/1 | 4/4 | - | - | - | 15/15 (100%) |
|  |  |  | Not published (m) | 6K | 1/1 | 1/1 | 1/1 | 1/1 | 1/1 | 1/1 | 1/1 | 1/1 | 1/1 | 1/1 | 1/1 | 4/4 | - | - | - | 15/15 (100%) |
| MAYV-specific PCR | In-house RT-qPCR | Species | Waggoner et al.(74) | 5'UTR/NSP1 | 1/1 | 1/1 | 1/1 | 1/1 | 1/1 | 1/1 | 1/1 | 1/1 | 1/1 | 1/1 | 1/1 | 3/4 | - | - | 1 | 14/15 (93%) |
|  |  |  | Long et al.(75) | E2 | 1/1 | 1/1 | 1/1 | 1/1 | 1/1 | 1/1 | 1/1 | 1/1 | 1/1 | 1/1 | 1/1 | 4/4 | - | - | - | 15/15 (100%) |
|  |  |  | Friedrich-Jänicke et al.(76) | NSP1 | 1/1 | 1/1 | 1/1 | 1/1 | 1/1 | 1/1 | 1/1 | 1/1 | 1/1 | 1/1 | 1/1 | 4/4 | - | - | - | 15/15 (100%) |
|  |  |  | Gomes Naveca et al.(77) | NSP1 | 1/1 | 1/1 | 1/1 | 1/1 | 1/1 | 1/1 | 1/1 | 1/1 | 1/1 | 1/1 | 1/1 | 4/4 | - | - | - | 15/15 (100%) |
|  |  |  | Not published (n) | NSP1 | 1/1 | 1/1 | 1/1 | 1/1 | 1/1 | 1/1 | 1/1 | 1/1 | 1/1 | 1/1 | 1/1 | 4/4 | - | - | - | 15/15 (100%) |
|  |  |  | Not published (o) | NSP1 | 1/1 | 1/1 | 1/1 | 1/1 | 1/1 | 1/1 | 1/1 | 1/1 | 1/1 | 1/1 | 1/1 | 4/4 | - | - | - | 15/15 (100%) |
|  |  |  | Not published (p) | E1 | 1/1 | 1/1 | 1/1 | 1/1 | 1/1 | 1/1 | 1/1 | 1/1 | 1/1 | 1/1 | 1/1 | 4/4 | - | - | - | 15/15 (100%) |
|  |  |  | Not published (q) | E2 | 1/1 | 1/1 | 1/1 | 1/1 | 1/1 | 1/1 | 1/1 | 1/1 | 1/1 | 0/1 | 1/1 | 4/4 | - | 1 | - | 14/15 (93%) |

| **Assay design** | **Method** | **Result level** | **Reference**  **or manufac-turer** | **Target** | **RRV** | **SINV** | **WEEV** | **VEEV** | **EEEV** | **BFV** | **MAYV** | **ONNV** | **CHIKV-Asian** | **CHIKV- West African** | **CHIKV-ECSA** | **Negative samples** | **False positive (FP)** | **False negative (FN)** | **Incon-clusive** | **Correct entries, n (%)** |
| --- | --- | --- | --- | --- | --- | --- | --- | --- | --- | --- | --- | --- | --- | --- | --- | --- | --- | --- | --- | --- |
| ONNV-specific PCR | In-house RT-qPCR | Species | Waggoner et al.(74) | 5'UTR-NSP1 | 2/2 | 2/2 | 2/2 | 2/2 | 2/2 | 2/2 | 2/2 | 1/2 | 2/2 | 2/2 | 2/2 | 8/8 | - | 1 | - | 14/15 (93%) |
|  |  |  | Not published (r) | NSP1 | 1/1 | 1/1 | 1/1 | 1/1 | 1/1 | 1/1 | 1/1 | 1/1 | 1/1 | 1/1 | 1/1 | 4/4 | - | - | - | 15/15 (100%) |
|  |  |  | Not published (s) | NSP2 | 1/1 | 1/1 | 1/1 | 1/1 | 1/1 | 1/1 | 1/1 | 1/1 | 1/1 | 1/1 | 1/1 | 4/4 | - | - | - | 15/15 (100%) |
|  |  |  | Not published (t) | NSP3 | 1/1 | 1/1 | 1/1 | 1/1 | 1/1 | 1/1 | 1/1 | 1/1 | 1/1 | 1/1 | 1/1 | 4/4 | - | - | - | 15/15 (100%) |
|  |  |  | Not published (u) | NSP1/NSP3 | 1/1 | 1/1 | 1/1 | 1/1 | 1/1 | 1/1 | 1/1 | 1/1 | 1/1 | 1/1 | 1/1 | 4/4 | - | - | - | 15/15 (100%) |
|  |  |  | Not published (v) | E1 | 1/1 | 1/1 | 1/1 | 1/1 | 1/1 | 1/1 | 1/1 | 1/1 | 1/1 | 1/1 | 1/1 | 4/4 | - | - | - | 15/15 (100%) |
|  |  |  | Not published (w) | Unknown | 1/1 | 1/1 | 1/1 | 1/1 | 1/1 | 1/1 | 1/1 | 0/1 | 1/1 | 1/1 | 1/1 | 4/4 | - | 1 | - | 14/15 (93%) |
| CHIKV-specifica PCR assay | In-house RT-qPCR | Species | Pastorino et al.(67) | E1 | 2/2 | 2/2 | 2/2 | 2/2 | 2/2 | 2/2 | 2/2 | 2/2 | 2/2 | 2/2 | 2/2 | 8/8 | - | - | - | 30/30 (100%) |
|  |  |  | Panning et al.(68) | NSP1 | 1/2 | 1/2 | 1/2 | 1/2 | 1/2 | 1/2 | 1/2 | 1/2 | 2/2 | 2/2 | 2/2 | 4/8 | 1 |  | 11 | 18/30 (60%) |
|  |  |  | Santiago et al.(69) | NSP1 | 2/2 | 2/2 | 2/2 | 2/2 | 2/2 | 2/2 | 2/2 | 2/2 | 2/2 | 2/2 | 2/2 | 8/8 | - | - | - | 30/30 (100%) |
|  |  |  | Waggoner et al.(70) | NSP2 | 1/1 | 1/1 | 1/1 | 1/1 | 1/1 | 1/1 | 1/1 | 1/1 | 1/1 | 1/1 | 1/1 | 4/4 | - | - | - | 15/15 (100%) |
|  |  |  | Thirion et al.(46) | E1-NSP1 | 1/1 | 1/1 | 1/1 | 1/1 | 1/1 | 1/1 | 1/1 | 1/1 | 1/1 | 1/1 | 1/1 | 4/4 | - | - | - | 15/15 (100%) |
|  |  |  | Lanciotti et al.(71) | NSP1 | 1/1 | 1/1 | 1/1 | 1/1 | 1/1 | 1/1 | 1/1 | 1/1 | 1/1 | 1/1 | 1/1 | 4/4 | - | - | - | 15/15 (100%) |
|  |  |  | Not published (x) | E1 | 1/1 | 1/1 | 1/1 | 1/1 | 1/1 | 1/1 | 1/1 | 1/1 | 1/1 | 1/1 | 1/1 | 4/4 | - | - | - | 15/15 (100%) |
|  |  |  | Not published (y) | E | 1/1 | 1/1 | 1/1 | 1/1 | 1/1 | 1/1 | 1/1 | 1/1 | 1/1 | 1/1 | 0/1 | 4/4 | - | 1 | - | 14/15 (93%) |

| **Assay design** | **Method** | **Result level** | **Reference  or manufac-turer** | **Target** | **RRV** | **SINV** | **WEEV** | **VEEV** | **EEEV** | **BFV** | **MAYV** | **ONNV** | **CHIKV-Asian** | **CHIKV- West African** | **CHIKV-ECSA** | **Negative samples** | **False positive (FP)** | **False negative (FN)** | **Incon-clusive** | **Correct entries, n (%)** |
| --- | --- | --- | --- | --- | --- | --- | --- | --- | --- | --- | --- | --- | --- | --- | --- | --- | --- | --- | --- | --- |
| CHIKV-specifica PCR assay | In-house RT-qPCR | Species | Not published (z) | NSP2 | 1/1 | 1/1 | 1/1 | 1/1 | 1/1 | 1/1 | 1/1 | 1/1 | 1/1 | 1/1 | 0/1 | 4/4 | - | 1 | - | 14/15 (93%) |
|  |  |  | Not published (aa) | NSP1-E1 | 1/1 | 1/1 | 1/1 | 1/1 | 1/1 | 1/1 | 1/1 | 1/1 | 1/1 | 1/1 | 1/1 | 4/4 | - | - | - | 15/15 (100%) |
|  |  |  | Not published (ab) | NSP1 | 1/1 | 1/1 | 1/1 | 1/1 | 1/1 | 1/1 | 1/1 | 1/1 | 1/1 | 1/1 | 1/1 | 4/4 | - | - | - | 15/15 (100%) |
|  |  |  | Not published (ac) | NSP1 | 1/1 | 1/1 | 1/1 | 1/1 | 1/1 | 1/1 | 1/1 | 0/1 | 1/1 | 1/1 | 1/1 | 4/4 | 1 | - | - | 14/15 (93%) |
|  |  |  | Not published (ad) | Unknown | 1/1 | 1/1 | 1/1 | 1/1 | 1/1 | 1/1 | 1/1 | 0/1 | 1/1 | 1/1 | 1/1 | 4/4 | 1 | - | - | 14/15 (93%) |
|  | Commercial RT-qPCR | Species | RealSTar Chikungunya RT-PCR kit v2.0, Altona Diagnostics ^c^ | Unknown | 4/4 | 5/5 | 5/5 | 5/5 | 5/5. | 4/4 | 4/4 | 2/4 | 4/4 | 5/5 | 4/4 | 20/20 | 2 | - | - | 67/69 (97%) |
|  |  |  | Quanty Chikungunya, Clonit | NSP2 | 1/1 | 1/1 | 1/1 | 1/1 | 0/1 | 1/1 | 1/1 | 0/1 | 1/1 | 1/1 | 0/1 | 4/4 | 2 | 1 | - | 12/15 (80%) |
|  |  |  | Chikungunya Light Mix, Roche ^f^ | E1 | 1/1 | 1/1 | 1/1 | 1/1 | 1/1 | 1/1 | 1/1 | 1/1 | 0/1 | 1/1 | 1/1 | 3/4 | - | 1 | 1 | 13/15 (87%) |
|  | In-house RT-PCR |  | Nyari et al.(78) | E1 | 1/1 | 1/1 | 1/1 | 1/1 | 1/1 | 1/1 | 1/1 | 1/1 | 1/1 | 0/1 | 1/1 | 4/4 | - | 1 | - | 14/15 (93%) |

The table reports results for each assay and each panel entry. Number correct/total number result entries; Incorrect results in red font; Incorrect results can be either false negative, false positive for a different alpha virus species, false positive for a negative sample or inconclusive.

^a^ Both laboratories that used this method to test the samples on genus level, also used additional other methods that could differentiate between the different samples.

^b^ Although these methods can be used to detect all alpha samples of this EQA, one laboratory used these methods only to analyze a subset of the EQA samples, as indicated by the total numbers per samples.

^C^ One laboratory used this method to analyze only a subset of the EQA samples, as indicated by the total number of tests per sample. They did not explain this deviation.
