# Supplementary material for: Low capacity for molecular detection of Alphaviruses other than Chikungunya virus in 23 European laboratories, March 2022
Source: PLoS One. 2025 Feb 27;20(2):e0318602. doi: 10.1371/journal.pone.0318602 (PMC11867335; doi:10.1371/journal.pone.0318602)
Supplement: Attachment — Submitted filename: RECOMMENDATIONS AND COMMENTS PONE-D-24-47893.docx [file pone.0318602.s003.docx]

**RECOMMENDATIONS AND COMMENTS**

**SUMMARY**

This study presents a timely and valuable evaluation of European laboratories' diagnostic capabilities for Alphaviruses, emphasizing the need to expand beyond the widely tested Chikungunya virus (CHIKV). Conducted in March 2022, the external quality assessment (EQA) involved 24 laboratories testing 15 samples representing nine Alphavirus species. Impressively, only 46% of the laboratories achieved full identification accuracy, indicating variability in diagnostic efficacy, especially for viruses like Venezuelan Equine Encephalitis Virus (VEEV) and Eastern Equine Encephalitis Virus (EEEV).

The strengths of this study include its comprehensive approach and inclusion of less commonly tested viruses, such as Ross River Virus (RRV) and Sindbis Virus (SINV), providing critical insights into current diagnostic limitations across Europe. However, the study also reveals significant gaps in Alphavirus detection sensitivity and specificity, especially outside of CHIKV. These findings underscore an urgent need for regular EQAs, improved diagnostic tools, and advanced training initiatives, especially in light of global climate shifts and increased vector migration that could expand Alphavirus distribution.

Overall, this research serves as an essential call to action for enhanced diagnostic capabilities and preparedness across Europe, laying a strong foundation for the continuous improvement of Alphavirus surveillance and control.

Minor Comment

1. Line 389 – Kindly insert the close Bracket for the open bracket if any

Major Comment

1. Line 56- I see a total of 15 samples which has been broken down to Nine alphavirus and 4 NCs. There are about 2 unknowns, Kindly account for that.
2. Line 89- What kind of other two mosquitoes? Kindly include
3. Figure one is kind of blur. If the image can be much sharper, it will be well appreciated.
4. Line 163 – It is best to please show the data or make it available by sharing the link to the repository. It is good you notified readers about data not been shown.
5. Line 185- Table 1. Kindly label the keys as keys and please space it a bit
6. Line 461- Contamination can also be a very key factor for false Negative and Positive results. Sample Mix up is great but does not hold weight. Makes it look like the lab is not up to task.

Others

1. Kindly include any limitations at the conclusion if any
