# Supplementary material for: Low capacity for molecular detection of Alphaviruses other than Chikungunya virus in 23 European laboratories, March 2022
Source: PLoS One. 2025 Feb 27;20(2):e0318602. doi: 10.1371/journal.pone.0318602 (PMC11867335; doi:10.1371/journal.pone.0318602)
Supplement: Attachment — Submitted filename: PONE-D-24-47893_Pezzi et al_comments.docx [file pone.0318602.s004.docx]

The study “Low capacity for molecular detection of Alphaviruses other than chikungunya virus in 24 European laboratories, March 2022” by Pezzi et al. aims to address an important concern to prevent the emergence of alphaviruses in the EU through regular assessment using EQA. The manuscript is well written, but has certain limitations, addressing which would make the paper more impactful.

a) Lines 51-52 & 139-141: Authors directly mention using EQA as the diagnostic technique, without giving an introduction and pros and cons of other diagnostic methods used in the field.
b) Lines 56-60: Authors mention 15 samples, but have listed 9 alphaviruses & 4 negative controls; what were the other 2 samples?
c) The selection of 24 laboratories in 16 European countries seems to aggregate to one particular region. Did the authors have a selection criterion for choosing these laboratories? Although the authors mention that not all the laboratories invited for the study agreed to participate, approaches to include more laboratories over a wider geographical range would be a better representation for the assessment techniques.
d) Line 131: Authors state that the vector competence of competent vectors for alphaviruses other than CHIKV is not yet known. However, MAYV circulation has been observed in Haiti ([Emergence of recombinant Mayaro virus strains from the Amazon basin | Scientific Reports](https://www.nature.com/articles/s41598-017-07152-5)) and *Aedes aegypti* has been shown to efficiently transmit MAYV under experimental conditions ([Dynamic of Mayaro Virus Transmission in Aedes aegypti, Culex quinquefasciatus Mosquitoes, and a Mice Model](https://www.mdpi.com/1999-4915/15/3/799)). Therefore, these considerations should be kept in mind for future EQAs.
e) This EQA was based on detection of viral RNA, however the presence of neutralizing antibodies would be a much better measure of viral infection and would be specific to the viruses as well. Future EQAs should focus on including this approach.
