# Supplementary material for: Low capacity for molecular detection of Alphaviruses other than Chikungunya virus in 23 European laboratories, March 2022
Source: PLoS One. 2025 Feb 27;20(2):e0318602. doi: 10.1371/journal.pone.0318602 (PMC11867335; doi:10.1371/journal.pone.0318602)
Supplement: Attachment — Submitted filename: Response to Reviewers.docx [file pone.0318602.s005.docx]

Response to Reviewers

PONE-D-24-47893

Low capacity for molecular detection of Alphaviruses other than Chikungunya virus in 24 European laboratories, March 2022

Journal requirements:

When submitting your revision, we need you to address these additional requirements.

1. Please ensure that your manuscript meets PLOS ONE's style requirements, including those for file naming. The PLOS ONE style templates can be found at

<https://journals.plos.org/plosone/s/file?id=wjVg/PLOSOne_formatting_sample_main_body.pdf> and

[https://journals.plos.org/plosone/s/file?id=ba62/PLOSOne_formatting_sample_title_authors_affiliations.pdf](https://track.editorialmanager.com/CL0/https:%2F%2Fjournals.plos.org%2Fplosone%2Fs%2Ffile%3Fid=ba62%2FPLOSOne_formatting_sample_title_authors_affiliations.pdf/1/010f01934f6836ba-f5a82c44-c167-4ea1-a03b-15bb8ec6826d-000000/ANXHoRcgTGgiB1RuxN5VpfBxxRWmhSNvSVoO_xWFMsI=186)

Author’s Response: We have reviewed and edited the manuscript according to the PLOS ONE style requirements (links above). Thank you for including them, they were very helpful. Apologies for not using/applying them completely for the initial submission.

2. Thank you for stating the following financial disclosure:  [This work was financed by the European Centre for Disease Prevention and Control (ECDC) under specific contract No 1 ECD.11886 ID 23729 implementing Framework contract No ECDC/2020/010. For providing access to the virus strains used, we thank the EVA-GLOBAL consortium (funded by the European Union’s Horizon 2020 research and innovation programme under grant agreement No. 871029). Except VEEV, WEEV and EEEV, the material was provided by the European virus archive-Marseille (EVAM) under the label technological platforms of Aix-Marseille University. CBEMR and RNC were both coordinators or work package leaders for ECDC and EU Horizon funding.].

Please state what role the funders took in the study.  If the funders had no role, please state: "The funders had no role in study design, data collection and analysis, decision to publish, or preparation of the manuscript."

If this statement is not correct you must amend it as needed.

Please include this amended Role of Funder statement in your cover letter; we will change the online submission form on your behalf.

Author’s Response: We have updated the financial disclosure with the following sentences regarding the “Role of the Funder”. “The funder had no role in study design other than final approval. The funder had no role in data collection and analysis, decision to publish, or preparation of the manuscript. However, the draft manuscript was submitted to ECDC for review and approval.

3. We note that your Data Availability Statement is currently as follows: [All relevant data are within the manuscript and its Supporting Information files.]

Please confirm at this time whether or not your submission contains all raw data required to replicate the results of your study. Authors must share the “minimal data set” for their submission. PLOS defines the minimal data set to consist of the data required to replicate all study findings reported in the article, as well as related metadata and methods (<https://journals.plos.org/plosone/s/data-availability#loc-minimal-data-set-definition>).

For example, authors should submit the following data:

- The values behind the means, standard deviations and other measures reported;

- The values used to build graphs;

- The points extracted from images for analysis.

Authors do not need to submit their entire data set if only a portion of the data was used in the reported study.

If your submission does not contain these data, please either upload them as Supporting Information files or deposit them to a stable, public repository and provide us with the relevant URLs, DOIs, or accession numbers. For a list of recommended repositories, please see <https://journals.plos.org/plosone/s/recommended-repositories>.

If there are ethical or legal restrictions on sharing a de-identified data set, please explain them in detail (e.g., data contain potentially sensitive information, data are owned by a third-party organization, etc.) and who has imposed them (e.g., an ethics committee). Please also provide contact information for a data access committee, ethics committee, or other institutional body to which data requests may be sent. If data are owned by a third party, please indicate how others may request data access.

Author’s Response: We will upload the de-identified raw data from the laboratories. We can refer to them as S1 Table and change the current S1 Table to the new S2 table.

4. We note that Figure 1 in your submission contain [map/satellite] images which may be copyrighted. All PLOS content is published under the Creative Commons Attribution License (CC BY 4.0), which means that the manuscript, images, and Supporting Information files will be freely available online, and any third party is permitted to access, download, copy, distribute, and use these materials in any way, even commercially, with proper attribution. For these reasons, we cannot publish previously copyrighted maps or satellite images created using proprietary data, such as Google software (Google Maps, Street View, and Earth). For more information, see our copyright guidelines: <http://journals.plos.org/plosone/s/licenses-and-copyright>.

Authors Response: There are no copyright issues with Figure 1. ECDC requires us to use their map making tool (EMMa) for their funded projects. However, as figures are created using EMMa and submitted in manuscripts, they are freely available to be published under CC BY 4.0.

Additional Editor Comments:

Dear Authors,

We are informing you that your manuscript has been reviewed and has received conflicting comments, as detailed in the email. My decision regarding this submission is to request a major revision. Please revise the manuscript according to the suggestions provided by all reviewers before it can be considered further for acceptance.


[Note: HTML markup is below. Please do not edit.]

Reviewers' comments:

Reviewer's Responses to Questions

**Comments to the Author**

1. Is the manuscript technically sound, and do the data support the conclusions?

The manuscript must describe a technically sound piece of scientific research with data that supports the conclusions. Experiments must have been conducted rigorously, with appropriate controls, replication, and sample sizes. The conclusions must be drawn appropriately based on the data presented.

Reviewer #1: Yes

Reviewer #2: Yes

Reviewer #3: Partly

2. Has the statistical analysis been performed appropriately and rigorously?

Reviewer #1: Yes

Reviewer #2: Yes

Reviewer #3: N/A

3. Have the authors made all data underlying the findings in their manuscript fully available?

The [PLOS Data policy](http://track.editorialmanager.com/CL0/http:%2F%2Fwww.plosone.org%2Fstatic%2Fpolicies.action%23sharing/1/010f01934f6836ba-f5a82c44-c167-4ea1-a03b-15bb8ec6826d-000000/3FjaiMx14Wl8yQNt03tSZk3Rv_A3TjW56ORh2w_pG5w=186) requires authors to make all data underlying the findings described in their manuscript fully available without restriction, with rare exception (please refer to the Data Availability Statement in the manuscript PDF file). The data should be provided as part of the manuscript or its supporting information, or deposited to a public repository. For example, in addition to summary statistics, the data points behind means, medians and variance measures should be available. If there are restrictions on publicly sharing data—e.g. participant privacy or use of data from a third party—those must be specified.

Reviewer #1: Yes

Reviewer #2: Yes

Reviewer #3: Yes

4. Is the manuscript presented in an intelligible fashion and written in standard English?

PLOS ONE does not copyedit accepted manuscripts, so the language in submitted articles must be clear, correct, and unambiguous. Any typographical or grammatical errors should be corrected at revision, so please note any specific errors here.

Reviewer #1: Yes

Reviewer #2: Yes

Reviewer #3: Yes

5. Review Comments to the Author

Please use the space provided to explain your answers to the questions above. You may also include additional comments for the author, including concerns about dual publication, research ethics, or publication ethics. (Please upload your review as an attachment if it exceeds 20,000 characters)

Reviewer #1: Submission well written containing all the fine details needed. The only issue is that there are some few misses that can be addressed as soon as the comments is released. Kindly address the comments as soon as possible. I enjoyed reviewing this paper because the English was not so difficult to comprehend therefor, anyone from varying background can read it.

Authors Response: Thank you for the kind words. We are happy to hear that the English was accessible and not overly complicated. We will certainly do our best to address all the reviewer and editor comments.

**RECOMMENDATIONS AND COMMENTS**

**SUMMARY**

This study presents a timely and valuable evaluation of European laboratories' diagnostic capabilities for Alphaviruses, emphasizing the need to expand beyond the widely tested Chikungunya virus (CHIKV). Conducted in March 2022, the external quality assessment (EQA) involved 24 laboratories testing 15 samples representing nine Alphavirus species. Impressively, only 46% of the laboratories achieved full identification accuracy, indicating variability in diagnostic efficacy, especially for viruses like Venezuelan Equine Encephalitis Virus (VEEV) and Eastern Equine Encephalitis Virus (EEEV).

The strengths of this study include its comprehensive approach and inclusion of less commonly tested viruses, such as Ross River Virus (RRV) and Sindbis Virus (SINV), providing critical insights into current diagnostic limitations across Europe. However, the study also reveals significant gaps in Alphavirus detection sensitivity and specificity, especially outside of CHIKV. These findings underscore an urgent need for regular EQAs, improved diagnostic tools, and advanced training initiatives, especially in light of global climate shifts and increased vector migration that could expand Alphavirus distribution.

Overall, this research serves as an essential call to action for enhanced diagnostic capabilities and preparedness across Europe, laying a strong foundation for the continuous improvement of Alphavirus surveillance and control.

Minor Comment

1. Line 389 – Kindly insert the close Bracket for the open bracket if any

Authors Response: We have now changed all brackets for references to the proper [closed bracket] rather than the (parentheses).

Major Comment

1. Line 56- I see a total of 15 samples which has been broken down to Nine alphavirus and 4 NCs. There are about 2 unknowns, Kindly account for that.

Authors Response: Table 3 shows the complete breakdown of all 15 samples included in the EQA panel. There are nine alphaviruses, however CHIKV has three different strains. So in the panel there are 15 total samples, 11 contain virus and 4 negative controls.

2. Line 89- What kind of other two mosquitoes? Kindly include

Authors Response: We added the most common mosquito vector genera (*Culex, Culiseta, Aedes, Coquillettidia,* and *Haemogogus*) and further species descriptions are specified for specific alphaviruses in the European context.

3. Figure one is kind of blur. If the image can be much sharper, it will be well appreciated.

Authors Response: Yes, unfortunately ECDC (our funder) requires us to use their online map making tool (EMMa). This is the highest resolution image that is offered by the tool. We will work with the editor and journal proofing staff to ensure the highest possible quality for all figures and images in the manuscript.

4. Line 163 – It is best to please show the data or make it available by sharing the link to the repository. It is good you notified readers about data not been shown.

Authors Response: Yes, we can share the raw data file with the journal. The participating laboratories will be de-identified and given a random number. Raw data file will be labeled as S1 Table and be referenced in the text of the manuscript approximately lines 165 and 222.

5. Line 185- Table 1. Kindly label the keys as keys and please space it a bit

Authors Response: Noted and edited.

6. Line 461- Contamination can also be a very key factor for false Negative and Positive results. Sample Mix up is great but does not hold weight. Makes it look like the lab is not up to task.

Authors Response: While we cannot rule out sample mix up, or wrong reporting for false positives, you are correct that contamination is also certain to be a possibility. It has been added to line 463.

Others

1. Kindly include any limitations at the conclusion if any

Authors Response: Limitations are listed as follows starting approximately line 484 “This European alphavirus EQA has its limitations. The EQA did not contain any challenging samples with very low viral concentrations. Although viral loads are typically high for some alphaviruses such as CHIKV, they can vary according to the phase of the disease, nature of the sample (i.e., blood, urine, saliva) and sample handling conditions. It is advised to address sensitivity of detection in more detail in future EQAs. Furthermore, this EQA assessed only molecular diagnosis capacity, which is typically performed in the first 5-7 days post symptoms onset. Assessing performances of serology tools is fundamental to fully evaluate diagnostic capacity for alphaviruses at the European level. However, serology EQA panels for alphaviruses are a more challenging, more expensive problem due to the need for positive (virus, IgG, and/or IgM) serum.

Reviewer #2: The manuscript evaluates the molecular detection capabilities of alphaviruses in expert laboratories across Europe through an external quality assessment (EQA) in March 2022. The study involved 24 laboratories from 16 countries and used a panel of 15 samples, including nine alphaviruses and four negative controls. The study found that only CHIKV and SINV are currently circulating in Europe. While molecular detection for these was satisfactory, the overall performance for the panel was below 50%. The authors recommend improving diagnostics for alphaviruses in Europe and conducting regular EQAs to enhance laboratory capabilities, especially with the potential impact of climate change and vector expansion.
Although the research is interesting, the study acknowledges limitations that may affect the generalizability of the results. Addressing these issues would enhance the study's relevance and suitability for publication.

Authors Response: Yes, we think it is important to acknowledge the limitations. Proficiency panel or External Quality Assessment panel design for diagnostics is a very important public health laboratory function. Design of these panels can be exceedingly complex (assessment of entire workflows from sampling to sample transport to sample storage, extraction, PCR, etc.) or quite simple (1-2 Ct values of an inactivated sample to assess the sensitivity and specificity of a PCR assay). For this EQA, due to funding and sometimes complicated international shipping regulations in EU and non-EU European countries, we have limits as to how complex we can make our EQA panels. Unfortunately, we are also primarily limited to molecular EQA panels, as serology EQAs would require positive serum (often not available for rare, emerging viruses, and often very complicated to ship/transport due to international regulations).

- The study mentions 24 laboratories as participants in the external quality assessment (EQA), but the results appear to reflect data from only 23 laboratories (24 laboratories registered for the assessment, only 23 contributed valid results). I recommend revising the title and abstract to avoid confusion and enhance clarity, ensuring the representation of the participant count is accurate.

Authors Response: Yes, excellent suggestion. I suspect 24 was written before the analysis was complete and then 1 lab had incomplete data and was dropped out resulting in 23. We have now changed the title and the abstract/introduction sentences that said 24 laboratories to now read 23 laboratories.

- Could you clarify the sample types used in the study (e.g., blood, urine, saliva)? This information would help to better understand the methodology.

Authors Response: Yes, the panel samples are human plasma spiked with inactivated virus. The process is described in “Panel preparation and validation” approximately line 533.

- The EQA did not include a wide range of viral loads or challenging samples, it may not fully represent the diagnostic capabilities of laboratories under various real-world conditions.

Authors Response: So, this is an excellent topic of discussion because EQA panels can have such a broad range of complexity. It is true, this EQA panel isn’t so complex. There are a number of related viruses here which can cause specificity issues, and the RNA copies/uL was chosen as it was meant to reflect approximate real-world values for each of these viruses. We could have created a much larger panel with a wider range of viral loads, some low copy number samples (some EQA providers call these “educational” samples) and challenged not only specificity, but really interrogated sensitivity.

Alternatively (and at our institute in the Netherlands, we did this for SARS-CoV-2 in our national EQA panels), we could design our molecular EQA to test all aspects of the diagnostics chain, including sample transport, storage, extraction, etc. This can be challenging to analyses though due to the high number of “workflows” (different staff, extraction methods, PCR platforms, assays all result in a different “workflow” which can result in a massive amount of analysis necessary).

The limiting factors that have the most impact are, however, money and what our funding source (ECDC) requests. Unfortunately, the budget for these activities are quite low and If we were to increase complexity of the panels, or increase the amount of analysis necessary, the funding would from ECDC would not be adequate. Therefore, we do have to prioritize what is possible for these EQAs vs. what we would like to do, or what the most thorough EQA panel would be.

- The study primarily assessed molecular diagnostic methods, which may overlook the performance of serological tests. I recommend that the authors discuss this limitation.

Authors Response: We fully agree that for these viruses, serological tests are crucial. This sentence: “Furthermore, this EQA assessed only molecular diagnosis capacity, which is typically performed in the first 5-7 days post symptoms onset. Assessing performances of serology tools is fundamental to fully evaluate diagnostic capacity for alphaviruses at European level.” Is approximately on line 490. However, creating and distributing EQA panels for serological diagnosis of alphaviruses is very challenging considering the need for positive serum (either virus positive, IgG or IgM), and shipping logistics and regulations.

Inserted “However, serology EQA panels for alphaviruses are a more challenging, more expensive problem due to the need for positive (virus, IgG, and/or IgM) serum.” at line 491.

Reviewer #3: The study “Low capacity for molecular detection of Alphaviruses other than chikungunya virus in 24 European laboratories, March 2022” by Pezzi et al. aims to address an important concern to prevent the emergence of alphaviruses in the EU through regular assessment using EQA. The manuscript is well written, but has certain limitations, addressing which would make the paper more impactful.
a) Lines 51-52 & 139-141: Authors directly mention using EQA as the diagnostic technique, without giving an introduction and pros and cons of other diagnostic methods used in the field.

Authors Response: External Quality Assessments are not specifically diagnostic techniques. They are used to assess the efficacy and functionality of diagnostic techniques. In this manuscript, we assess the efficacy and functionality of molecular diagnostic tests used in 23 laboratories. For molecular and serology assays, we discuss pros and cons of each (for example, line 491).

b) Lines 56-60: Authors mention 15 samples, but have listed 9 alphaviruses & 4 negative controls; what were the other 2 samples?

Authors Response: Table 3 shows the complete breakdown of all 15 samples included in the EQA panel. There are nine alphaviruses, however CHIKV has three different strains. So in the panel there are 15 samples, 11 contain virus and 4 negative controls.

c) The selection of 24 laboratories in 16 European countries seems to aggregate to one particular region. Did the authors have a selection criterion for choosing these laboratories? Although the authors mention that not all the laboratories invited for the study agreed to participate, approaches to include more laboratories over a wider geographical range would be a better representation for the assessment techniques.

Authors Response: This EQA was funded by the European Centre for Disease Prevention and Control (ECDC). Therefore, only EU/EEA countries were allowed to participate/be invited to participate. All EU/EEA countries had institutes (or, in most cases, multiple institutes) that were invited. In the end, we had 23 total laboratories in EU/EEA countries respond. However, we completely agree with you that including more laboratories over a wider geographical range would be better for multiple reasons. This laboratory network (funded by ECDC) will evolve over the next few years into something a bit different (mentioned in the paper, the new EURL system). In the future, either through the new EURL system, or contacts with WHO Euro Region (includes many more countries than EU/EEA), we do hope (and are actively trying) to expand our geographical range for these panels.

d) Line 131: Authors state that the vector competence of competent vectors for alphaviruses other than CHIKV is not yet known. However, MAYV circulation has been observed in Haiti (Emergence of recombinant Mayaro virus strains from the Amazon basin | Scientific Reports) and Aedes aegypti has been shown to efficiently transmit MAYV under experimental conditions (Dynamic of Mayaro Virus Transmission in Aedes aegypti, Culex quinquefasciatus Mosquitoes, and a Mice Model). Therefore, these considerations should be kept in mind for future EQAs.

Authors Response: Indeed, for these alphaviruses, globally some vector competence is understood, and the research continues with new updates. The recent emergence of oropouche virus and imported cases into Europe have also led to updated consideration given to vector competence of some mosquito viruses. Thank you for the references regarding MAYV, the references have been added and updated.

e) This EQA was based on detection of viral RNA, however the presence of neutralizing antibodies would be a much better measure of viral infection and would be specific to the viruses as well. Future EQAs should focus on including this approach.

Authors Response: Assessing performances of serology tools is fundamental to fully evaluate diagnostic capacity for alphaviruses at the European level. However, serology EQA panels for alphaviruses are a more challenging, more expensive problem due to the need for positive (virus, IgG, and/or IgM) serum.

Performing neutralizing antibody tests would be an interesting EQA. There are very few laboratories that perform neutralizing antibody tests for these viruses in Europe. Of course, in many cases neutralization assays are the gold standard and are more specific than ELISAs or molecular assays.

It is an interesting suggestion. Most laboratories in Europe that have diagnostic capabilities rely on PCR and ELISAs for diagnostics (though sequencing is also important in some cases). However, there are still some laboratories that perform neutralization assays for some viruses and it would be interesting to do a comparison with proficiency panels.

While revising your submission, please upload your figure files to the Preflight Analysis and Conversion Engine (PACE) digital diagnostic tool, [https://pacev2.apexcovantage.com/](https://track.editorialmanager.com/CL0/https:%2F%2Fpacev2.apexcovantage.com%2F/1/010f01934f6836ba-f5a82c44-c167-4ea1-a03b-15bb8ec6826d-000000/KYbWef2Dj6piktRb4qxenJBQ8FtJ5shWZ8AACfk_CSA=186). PACE helps ensure that figures meet PLOS requirements. To use PACE, you must first register as a user. Registration is free. Then, login and navigate to the UPLOAD tab, where you will find detailed instructions on how to use the tool. If you encounter any issues or have any questions when using PACE, please email PLOS at [figures@plos.org](mailto:figures@plos.org). Please note that Supporting Information files do not need this step.
